# Prognostic evaluation of preoperative serum tumor marker‐negative cases in non‐small cell lung cancer: A retrospective study

**DOI:** 10.1002/cnr2.1696

**Published:** 2022-08-12

**Authors:** Yuichiro Onuki, Hirochika Matsubara, Ryunosuke Koizumi, Mamoru Muto, Harunobu Sasanuma, Daisuke Sato, Aya Sugimura, Tsuyoshi Uchida, Hiroyasu Matsuoka, Hiroyuki Nakajima

**Affiliations:** ^1^ Division of General Thoracic Surgery, Department of Surgery Yamanashi University Yamanashi Japan; ^2^ Department of Surgery Kofu Municipal Hospital Yamanashi Japan

**Keywords:** biology, lung cancer, prognosis, surgery, tumor markers

## Abstract

**Background:**

The role of various serum tumor markers (TMs) has been reported in non‐small cell lung cancer (NSCLC). However, the prognosis of patients with multiple TM‐negative NSCLC remain unclear.

**Aims:**

This study aimed to describe the characteristics and outcomes of patients with NSCLC undergoing surgery and to investigate their prognostic association with preoperative serum TM‐negative cases.

**Methods and results:**

We retrospectively evaluated 442 patients who underwent complete resection of stage I NSCLC between January 2004 and December 2019. These 442 patients were classified into a group whose preoperative serum levels of carcinoembryonic antigen (CEA), cytokeratin‐19 fragment (CYFRA21‐1), carbohydrate antigen 19‐9 (CA19‐9), and squamous cell carcinoma antigen (SCC Ag) were all negative (TM‐negative group; *n* = 249, 56%) and a group with at least one positive marker (TM‐positive group; *n* = 193, 44%). Among all patients, the TM‐negative group showed higher 5‐year recurrence‐free survival (RFS) (92.6% vs. 79.1%; *p* < .01), and overall survival (OS) rates (86.3% vs. 68.6%; *p* < .01). After propensity score matching, patients in the TM‐negative group still exhibited good 5‐year RFS (92.1% vs. 81.4%; *p* = .01) and OS rates (87.6% vs. 72.6%; *p* < .01).

**Conclusion:**

Our study suggests that NSCLC patients who are preoperatively negative for all serum TMs, such as CEA, CYFRA21‐1, CA19‐9, and SCC Ag, represent a subgroup with a particularly good prognosis.

## 1 INTRODUCTION

Lung cancer is the leading cause of cancer‐related deaths.^1^ In 2021, the American Cancer Society estimated that the prevalence of lung cancer is the second highest among all cancer types, and almost one‐quarter of all cancer‐related deaths are due to lung cancer.^2^ Early diagnosis of recurrence after surgery for lung cancer contributes to an improved prognosis. The role of various serum tumor markers (TMs) has been reported in non‐small cell lung cancer (NSCLC), but their efficiency in early diagnosis is limited. High preoperative serum levels of several TMs are associated with a poor prognosis in patients with NSCLC. In particular, the preoperative value of carcinoembryonic antigen (CEA) and cytokeratin‐19 fragment (CYFRA21‐1) may provide prognostic and predictive information for both recurrence and mortality risk in NSCLC.^3^, ^4^, ^5^, ^6^, ^7^, ^8^ Carbohydrate antigen 19‐9 (CA19‐9) is widely used to predict prognosis in patients with colorectal or pancreatic cancer. Accumulated evidence suggests a high positivity rate in lung adenocarcinoma as well.^9^ In addition, squamous cell carcinoma antigen (SCC Ag) is the most used prognostic marker for lung SCC.^10^, ^11^ The combined use of multiple TMs with relevant clinical factors, such as age or sex, may increase their prognostic accuracy; however, the results obtained using this approach have been inconsistent.^12^, ^13^, ^14^ The association between elevated preoperative serum TM levels and prognosis is well known. However, the prognosis of patients with multiple TM‐negative NSCLC remains unclear. It is important to investigate these patients as they are easy to follow‐up and may possibly represent a subgroup with a particularly good prognosis.

This study aimed to describe the characteristics and outcomes of patients with NSCLC undergoing surgery and to examine the prognosis in preoperatively TM‐negative patients.

## 2 METHODS

### 2.1 Patients and study design

We conducted a retrospective study data of patients who underwent surgery for NSCLC between January 2004 and December 2019. We reviewed the medical records of patients to examine their socio‐demographic profiles (age, sex, and smoking history), clinical status (comorbid chronic obstructive pulmonary disease or interstitial pneumonia), surgical treatment, tumor characteristics (pathological tumor size, histological subclassification, lymphovascular invasion, visceral pleural invasion, pathological stage, mutation status of epidermal growth factor receptor [EGFR]), adjuvant therapy (chemotherapy and/or radiation), and preoperative serum CEA, CYFRA21‐1, CA19‐9, and SCC Ag levels. Pathological stage was determined according to the eighth edition of the International Tumor Node Metastasis (TNM) staging system.^15^ Thus, pathological tumor size was defined as the largest dimension of the invasive portion.

**FIGURE 1:**
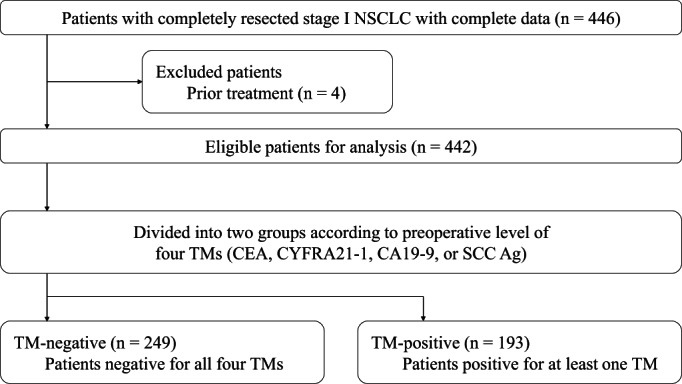
Study cohort flowchart. CA19‐9, carbohydrate antigen 19‐9; CEA, carcinoembryonic antigen; CYFRA21‐1, cytokeratin‐19 fragment; NSCLC, non‐small cell lung cancer; SCC Ag, squamous cell carcinoma antigen; TM, tumor marker

We included patients with completely resected stage I NSCLC and with complete data of preoperative serum TM levels (CEA, CYFRA21‐1, CA19‐9, and SCC Ag) (*n* = 446). Patients who had received prior induction or definitive treatment were excluded from the study (*n* = 4). Ultimately, 442 patients were classified into a group whose preoperative serum levels of CEA, CYFRA21‐1, CA19‐9, and SCC Ag were all negative (TM‐negative group; *n* = 249, 56%) or a group with at least one positive marker (TM‐positive group; *n* = 193, 44%) (Figure 1).

Patients were generally followed up every 3 months for the first 2 years after surgery and every 6–12 months thereafter. Computed tomography (CT) was performed every 6 months for the first 2 years after surgery and every 12 months after that. Additional examinations were also performed when the related symptoms occurred. Recurrent disease in the study was examined based on combined pathological examination and imaging evidence of CT or positron emission tomography (PET)‐CT and was confirmed by a radiologist and thoracic surgeon. This study was approved by the Institutional Review Board of the Yamanashi University Hospital (approval No. 2506). Informed consent was obtained in the form of opt‐out on the website (https://www.med.yamanashi.ac.jp/rinri/ippan.html) by the decision of the Institutional Review Board.

### 2.2 TM assays

We assessed four TMs, that is, CEA, CYFRA21‐1, CA19‐9, SCC Ag. Blood samples for TM measurements were obtained at least 1 month before surgery. To analyze the correlation between the preoperative TM levels and recurrence, TM levels were measured using an electrochemiluminescence immunoassay on the Cobas8000/e801® module (Roche Diagnostics, Pleasanton, CA, United States of America K.K., Tokyo, Japan). According to manufacturer's instructions, the cut‐off values were as follows: CEA, 5 ng/ml; CYFRA21‐1, 2.8 ng/ml; CA19‐9, 37 U/ml; and SCC Ag, 2.5 ng/ml. Any individual TM levels below the cut‐off value were defined as negative. We considered patients who were negative for all four TMs as “TM‐negative.”

### 2.3 Endpoints

The primary study endpoint was recurrence‐free survival (RFS), and the secondary study endpoint was overall survival (OS) after surgery in both the TM‐negative and TM‐positive groups.

### 2.4 Statistical analyses

Categorical and continuous variables were compared between the groups using Fisher's exact test or Pearson's chi‐square test and Student's *t* test, respectively. We estimated the survival rate using the Kaplan–Meier method and examined differences between groups using the log‐rank test. Furthermore, propensity score matching analysis was used to balance the characteristics of each group. The groups were adjusted through 1:1 matching, which was performed based on a logistic regression model that included clinicopathological factors, such as age, sex, smoking history, preoperative comorbidities, surgical treatment, histological subclassification, lymphovascular invasion, pathological stage, and adjuvant therapy. The identified caliper value was set at 0.2. All *p* values were two‐sided tested, and a *p* value less than .05 was considered statistically significant. All statistical analyses were performed using EZR version 1.54 (Saitama Medical Center, Jichi Medical University, Saitama, Japan), which is a graphical user interface for R version 4.03 (The R Foundation for Statistical Computing, Vienna, Austria).^16^

## 3 RESULTS

Table 1 shows the preoperative positive rate of each TM. CEA and CA19‐19 positivity gradually increased with increasing cancer stage, even in stages IA1 to IB. The baseline characteristics of patients in the TM‐negative and TM positive groups are summarized in Table 2. The average follow‐up period was 50.4 and 43.8 months in the TM‐negative and TM positive group, respectively. Compared with the TM‐positive group, the TM‐negative group comprised patients who were younger (*p* < .001), had fewer preoperative comorbidities (chronic obstructive pulmonary disease [*p* = .025] and interstitial pneumonia [*p* = .002]), and had smaller tumors (*p* < .001), as well as fewer smokers (*p* = .001). In addition, adenocarcinoma (*p* < .001) and early pathological stages (*p* = .001) were more common; while lymphovascular invasion (*p* < .001), visceral pleural invasion (*p* = .001), and EGFR expression (*p* < .001) were less common. Postoperative recurrence was more common in the TM‐positive group (15 patients [6.0%] vs. 34 patients [17.6%]).

**TABLE 1:** Preoperative positive rates of tumor markers

Variables	Pathological stage (*n* = 442)				
	IA1 (*n* = 154)	IA2 (*n* = 124)	IA3 (*n* = 45)	IB (*n* = 119)	All (*n* = 442)
CEA	17 (11.0)	22 (17.7)	13 (28.9)	38 (31.9)	90 (20.4)
CYFRA21‐1	33 (21.4)	33 (26.6)	12 (26.7)	45 (37.8)	123 (27.8)
CA19‐9	12 (7.8)	5 (4.0)	6 (13.3)	10 (8.4)	33 (7.5)
SCC Ag	12 (7.8)	3 (2.4)	2 (4.4)	13 (10.9)	30 (6.8)

*Note*: Values are presented as *n* (%).

Abbreviations: CA19‐9, carbohydrate antigen 19‐9; CEA, carcinoembryonic antigen; CYFRA21‐1, cytokeratin‐19 fragment; SCC Ag, squamous cell carcinoma antigen.

**TABLE 2:** Baseline characteristics of the overall cohort

Variable	TM‐negative (*n* = 249)	TM‐positive (*n* = 193)	*p* Value
Sex			.203
Male	143 (57.4)	123 (63.7)	
Female	106 (42.6)	70 (36.3)	
Age (years)	68.6 (±8.6)	71.2 (±7.5)	<.001
Follow‐up period (months)	50.4 (±31.4)	43.8 (±31.7)	.031
Smoking history			.001
Yes	140 (56.2)	138 (71.5)	
No	109 (43.8)	55 (28.5)	
Preoperative comorbidity			
COPD	70 (28.1)	74 (38.3)	.025
IP	18 (7.2)	33 (17.1)	.002
Extent of resection			.261
Pneumonectomy	1 (0.4)	2 (1.0)	
Lobectomy	183 (73.5)	128 (66.3)	
Segmentectomy	39 (15.7)	43 (22.3)	
Wedge resection	26 (10.4)	20 (10.4)	
Pathological tumor size (cm)	1.5 (±0.9)	1.8 (±1.1)	<.001
Tumor histology			<.001
Adenocarcinoma	206 (82.7)	127 (65.8)	
Squamous cell carcinoma	34 (13.7)	48 (24.9)	
Others	9 (3.6)	18 (9.3)	
Lymphovascular invasion			<.001
Yes	50 (20.1)	70 (36.3)	
No	194 (77.9)	119 (61.7)	
Not available	5 (2.0)	4 (2.1)	
Visceral pleural invasion			.001
Yes	38 (15.3)	54 (28.0)	
No	211 (84.7)	139 (72.0)	
Pathological stage			.005
IA1	98 (39.4)	56 (29.0)	
IA2	75 (30.1)	49 (25.4)	
IA3	25 (10.0)	20 (10.4)	
IB	51 (20.5)	68 (35.2)	
EGFR mutation			<.001
Yes	108 (43.4)	51 (26.4)	
No	123 (49.4)	125 (64.8)	
Not available	18 (7.2)	17 (8.8)	
Adjuvant therapy			.102
Yes	19 (7.6)	7 (3.6)	
No	230 (92.4)	186 (96.4)	
Postoperative recurrence	15 (6.0)	34 (17.6)	<.001
Preoperative TM levels			
CEA (ng/ml)	2.4 (±1.0)	7.4 (±19.5)	<.001
CYFRA21‐1 (U/ml)	1.7 (±0.5)	3.6 (±2.2)	<.001
CA19‐9 (ng/ml)	11.3 (±7.3)	34.2 (±35.0)	.008
SCC Ag (ng/ml)	1.0 (±0.4)	1.8 (±4.4)	.003

*Note*: Values are presented as *n* (%) or means (±SDs).

Abbreviations: CA19‐9, carbohydrate antigen 19‐9; CEA, carcinoembryonic antigen; COPD, chronic obstructive pulmonary disease; CYFRA21‐1, cytokeratin‐19 fragment; EGFR, epidermal growth factor receptor; IP, interstitial pneumonia; SCC Ag, squamous cell carcinoma antigen; TM, tumor marker.

Next, we performed a 1:1 propensity score matching analysis. The baseline characteristics of the matched stage I NSCLC patients are listed in Table 3. There were no statistically significant differences in clinicopathological factors, except preoperative recurrences and TM levels, between the two groups. On the other hand, preoperative TM levels of CEA, CYFRA21‐1, CA19‐9, and SCC Ag remained significantly different.

**TABLE 3:** Baseline characteristics of propensity score matched pairs

Variable	Propensity score matched pairs		
	TM‐negative (*n* = 150)	TM‐positive (*n* = 150)	*p* Value
Sex			˃.999
Male	92 (61.3)	93 (62.0)	
Female	58 (38.7)	57 (38.0)	
Age (years)	70.4 (±8.1)	70.3 (±7.5)	.900
Follow‐up period (months)	46.4 (±27.5)	47.3 (±33.2)	.803
Smoking history			.904
Yes	96 (64.0)	98 (65.3)	
No	54 (36.0)	52 (34.7)	
Preoperative comorbidity			
COPD	53 (35.3)	47 (31.3)	.540
IP	13 (8.7)	14 (9.3)	˃.999
Extent of resection			.951
Pneumonectomy	1 (0.7)	1 (0.7)	
Lobectomy	102 (68.0)	106 (70.7)	
Segmentectomy	29 (19.3)	26 (17.3)	
Wedge resection	18 (12.0)	17 (11.3)	
Pathological tumor size (cm)	1.5 (±1.0)	1.7 (±1.0)	.214
Tumor histology			.891
Adenocarcinoma	115 (76.7)	115 (76.7)	
Squamous cell carcinoma	27 (18.0)	25 (16.7)	
Others	8 (5.3)	10 (6.7)	
Lymphovascular invasion			.629
Yes	38 (25.3)	42 (28.0)	
No	112 (74.7)	108 (72.0)	
Not available	5 (2.0)	4 (2.1)	
Visceral pleural invasion			˃.999
Yes	29 (19.3)	30 (20.0)	
No	121 (80.7)	120 (80.0)	
Pathological stage			.867
IA1	58 (38.7)	52 (34.7)	
IA2	40 (26.7)	43 (28.7)	
IA3	13 (8.7)	16 (10.7)	
IB	39 (26.0)	39 (26.0)	
EGFR mutation			.533
Yes	54 (36.0)	46 (30.7)	
No	89 (59.3)	90 (60.0)	
Not available	7 (4.7)	14 (9.3)	
Adjuvant therapy			˃.999
Yes	6 (4.0)	7 (4.7)	
No	144 (96.0)	186 (95.3)	
Postoperative recurrence	10 (6.7)	24 (16.0)	.017
Preoperative TM levels			
CEA (ng/ml)	2.6 (±1.1)	7.9 (±22.0)	.003
CYFRA21‐1 (U/ml)	1.8 (±0.5)	3.3 (±1.8)	<.001
CA19‐9 (ng/ml)	11.6 (±0.4)	37.2 (±152.2)	.041
SCC Ag (ng/ml)	1.0 (±0.4)	1.8 (±4.9)	.045

*Note*: Values are presented as *n* (%) or means (±SDs).

Abbreviations: CA19‐9, carbohydrate antigen 19‐9; CEA, carcinoembryonic antigen; COPD, chronic obstructive pulmonary disease; CYFRA21‐1, cytokeratin‐19 fragment; EGFR, epidermal growth factor receptor; IP, interstitial pneumonia; SCC Ag, squamous cell carcinoma antigen; TM, tumor marker.

### 3.1 Survival analyses

**FIGURE 2:**
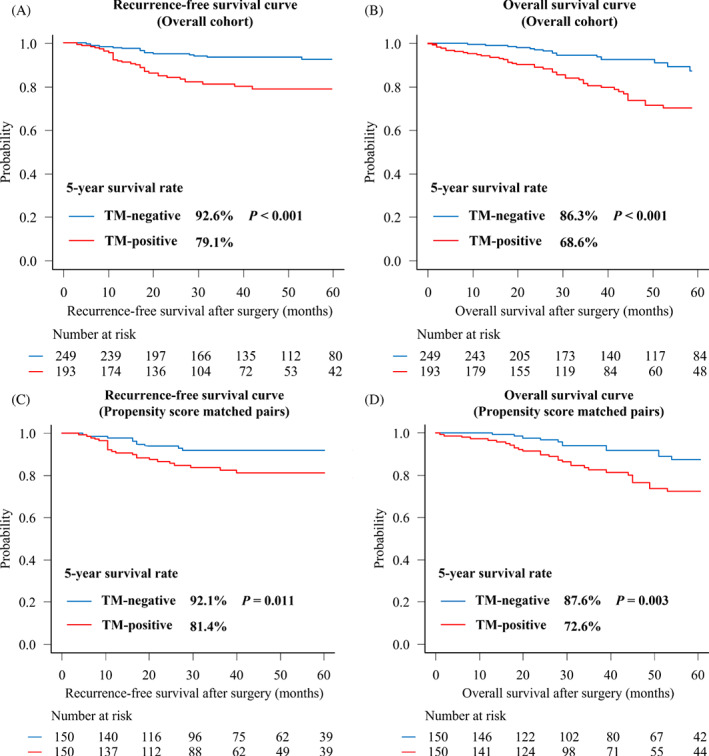
Kaplan–Meier curves for stage I non‐small cell lung cancer according to preoperative tumor marker (TM) levels (TM‐negative; blue, TM‐positive; red). (A) Recurrence‐free survival (RFS) and (B) overall survival curves for overall patients, (C) RFS, and (D) OS curves after propensity score matching

In the unmatched cohort, the 5‐year RFS (92.6% vs. 79.1%; *p* < .01) and OS (86.3% vs. 68.6%; *p* < .01) rates showed significant differences between the two groups (Figure 2A,B). After propensity score matching, the patients in the TM‐negative group still exhibited improved 5‐year RFS (92.1% vs. 81.4%; *p* = .01) and OS (87.6% vs. 72.6%; *p* < .01) rates (Figure 2C,D). These data suggested that combined negativity of preoperative serum TMs, such as CEA, CYFRA21‐1, CA19‐9, and SCC Ag, is an independent prognostic factor.

## 4 DISCUSSION

Although many biomarkers for lung cancer have been identified,^17^, ^18^ several serum TMs are used more widely, and the use of such assays is minimally invasive, convenient, and relatively inexpensive in clinical practice. However, the clinical significance of serum TM measurement remains controversial.^19^, ^20^ The overall TM positivity rates were lower in our study than in previous studies (Table 1). For example, studies that also included patients with stage I–III completely resected NSCLC^19^, ^21^ reported preoperative serum CEA levels of 33%–38%, compared with the 20.4% reported in our study focused on stage I patients. This finding could be explained by the high rate of surgery for early‐stage lung cancer. In general, serum TM levels gradually increase as lung cancer progresses.^3^, ^22^, ^23^, ^24^ In contrast, the frequency of surgery for early‐stage lung cancer is increasing, especially in Japan.^25^, ^26^ In 2017, stage I lung cancer accounted for 70.9% of invasive lung cancer surgeries, according to a report by the Japanese Association for Thoracic Surgery.^25^ The most recently reported preoperative TM levels are possibly lower than the levels reported previously.^27^, ^28^ In patients with elevated preoperative TM levels, re‐elevation of postoperative TM levels could help rule out recurrence. However, in patients with TM‐negative lung cancer, it can be considered as a predictor of prognosis.

Identifying EGFR mutations that may be the target of molecular therapy is crucial for NSCLC treatment. In fact, the use of tyrosine kinase inhibitors dramatically improves RFS and OS, especially in patients with advanced NSCLC. The previous studies reported an association between the levels of TMs, such as CEA, CYFRA21‐1, CA19‐9, and SCC Ag, and the rate of EGFR mutations.^28^, ^29^ More specifically, the rate of EGFR mutations increases in proportion to CEA levels. Demographic analyses have shown that a high prevalence of EGFR mutation is observed in women, nonsmokers, East Asian populations, and patients with adenocarcinoma,^30^, ^31^ which is consistent with the findings of our study. Our TM‐negative group had greater rates of EGFR mutations (43.4% vs. 26.4%) probably because of the predominance of nonsmokers and adenocarcinoma, as well as early‐stage lung cancer. However, after propensity score matching, there was no difference in the characteristics of patients with EGFR mutation, and EGFR status did not affect prognosis.

Regarding follow‐up methods after lung cancer surgery, the European Society for Medical Oncology guidelines recommend that medical history be monitored and physical examination performed every 6 months for 2 years after surgery when a period of relatively high recurrence rate is observed, with regular annual consultation thereafter.^32^ In addition, it is recommended that contrast‐enhanced CT be performed at least 12 and 24 months after surgery. The American Society of Clinical Oncology also recommends surveillance via a clinical examination (including CT) every 6 months for 2 years after surgery but does not recommend TM measurement for surveillance.^33^ In our study, pathological tumor size, visceral pleural invasion, and lymphovascular invasion, which are known prognostic factors in lung cancer, were significantly greater in the TM‐positive group. Although it would be complicated to try and to predict the prognosis by combining these factors, it may be possible to simplify postoperative follow‐up by classifying stage I NSCLC patients into TM‐negative and TM‐positive groups.

This study has some limitations. First, our study was not based on multicenter cohort data, which complicates the generalizability of our findings. Second, we limited ourselves to the examination of only four TMs, which are routinely measured at our institution. Further clinical studies are needed to assess the prognostic importance of other markers in TM‐negative cases.

In conclusion, our study suggests that patients with NSCLC who are preoperatively negative for all serum TMs, such as CEA, CYFRA21‐1, CA19‐9, and SCC Ag, represent a subgroup with a particularly good prognosis.

## AUTHOR CONTRIBUTIONS

**Yuichiro Onuki:** Conceptualization (equal); data curation (equal); formal analysis (equal); investigation (equal); methodology (equal); validation (equal); visualization (equal); writing – original draft (equal); writing – review and editing (equal). **Hirochika Matsubara:** Conceptualization (equal); methodology (equal); supervision (equal); validation (equal); writing – review and editing (equal). **Ryunosuke Koizumi:** Data curation (equal); resources (equal). **Mamoru Muto:** Data curation (equal); resources (equal). **Harunobu Sasanuma:** Data curation (equal); resources (equal). **Daisuke Sato:** Data curation (equal); validation (equal). **Aya Sugimura:** Data curation (equal); validation (equal). **Tsuyoshi Uchida:** Data curation (equal); validation (equal). **Hiroyasu Matsuoka:** Data curation (equal); software (equal). **Hiroyuki Nakajima:** Project administration (equal); supervision (equal); writing – review and editing (equal).

## CONFLICT OF INTEREST

The authors have stated explicitly that there are no conflicts of interest in connection with this article.

## ETHICS STATEMENT

This study was approved by the Institutional Review Board of the Yamanashi University Hospital (Approval No. 2506).

## PATIENT CONSENT STATEMENT

Informed consent was obtained in the form of opt‐out on the website (https://www.med.yamanashi.ac.jp/rinri/ippan.html) by the decision of the Institutional Review Board.

## Data Availability

The datasets used and/or analyzed during the current study are available from the corresponding author on reasonable request.
